# An ethnographic exploration of influences on prescribing in general practice: why is there variation in prescribing practices?

**DOI:** 10.1186/1748-5908-8-72

**Published:** 2013-06-21

**Authors:** Aileen Grant, Frank Sullivan, Jon Dowell

**Affiliations:** 1 Population Health Sciences, University of Dundee, Mackenzie Building, Kirsty Semple Way, Dundee, Scotland, DD2 4BF, UK

**Keywords:** Prescribing, Quality, General practice, Primary care, Ethnographic, Qualitative

## Abstract

**Background:**

Prescribing is a core activity for general practitioners, yet significant variation in the quality of prescribing has been reported. This suggests there may be room for improvement in the application of the current best research evidence. There has been substantial investment in technologies and interventions to address this issue, but effect sizes so far have been small to moderate. This suggests that prescribing is a decision-making process that is not sufficiently understood. By understanding more about prescribing processes and the implementation of research evidence, variation may more easily be understood and more effective interventions proposed.

**Methods:**

An ethnographic study in three Scottish general practices with diverse organizational characteristics. Practices were ranked by their performance against Audit Scotland prescribing quality indicators, incorporating established best research evidence. Two practices of high prescribing quality and one practice of low prescribing quality were recruited. Participant observation, formal and informal interviews, and a review of practice documentation were employed.

**Results:**

Practices ranked as high prescribing quality consistently made and applied macro and micro prescribing decisions, whereas the low-ranking practice only made micro prescribing decisions. Macro prescribing decisions were collective, policy decisions made considering research evidence in light of the average patient, one disease, condition, or drug. Micro prescribing decisions were made in consultation with the patient considering their views, preferences, circumstances and other conditions (if necessary).

Although micro prescribing can operate independently, the implementation of evidence-based, quality prescribing was attributable to an interdependent relationship. Macro prescribing policy enabled prescribing decisions to be based on scientific evidence and applied consistently where possible. Ultimately, this influenced prescribing decisions that occur at the micro level in consultation with patients.

**Conclusion:**

General practitioners in the higher prescribing quality practices made two different ‘types’ of prescribing decision; macro and micro. Macro prescribing informs micro prescribing and without a macro basis to draw upon the low-ranked practice had no effective mechanism to engage with, reflect on and implement relevant evidence. Practices that recognize these two levels of decision making about prescribing are more likely to be able to implement higher quality evidence.

## Background

Prescribing medication is a core activity for general practitioners (GPs), and the four-fold variation identified in the quality and safety of prescribing suggests there may be substantial room for improvement [1-5]. The quality of prescribing is determined by using established prescribing measures and applying these to practice data.

Because the passive dissemination of research evidence has proven inadequate to improve the quality of prescribing [6,7], there has been substantial investment in a wide range of technologies and interventions designed to address this issue [8,9]. The extensive literature on initiatives to encourage the application of research evidence and change professional practice has shown the effects of these interventions are small to moderate [10-13]. When it comes to changing professional practice there are no ‘magic bullets’ [14], and multiple factors are involved in GPs decisions to change their prescribing habits [15]. It is recognized multi-faceted interventions are more likely to improve the quality of prescribing, yet we still know little about why [1,16].

The problem of changing clinicians prescribing behavior has also been explored by a number of qualitative studies. These have primarily focused on the influences on the prescribing of new drugs [1,17-19], the influence of managerial forms of control [20-22], the influence of specialists [23], the influence of patients [24-29], and little change in prescribing as defence of clinical autonomy [20,21,30,31]. It is recognized there are multiple sources of influence on GPs prescribing behavior [1,18,32] but we do not know how these influences become embedded into routine practice.

There has been widespread interest in managing organizational culture within the UK National Health Service (NHS) to improve quality and safety [33,34], which focuses on organizations under direct NHS control such as hospitals [35-37] or primary care trusts [33,38], rather than general practices that are independently managed [39]. Previous studies using ethnographic methods have explored the culture of general practice in relation to financial incentives [40], knowledge management [41], and the new General Medical Services (nGMS) contract [42-44]. A holistic approach to understanding the influences GPs recognize when prescribing is relatively unexplored and findings from qualitative research are needed to better understand prescribing in general practice [45].

Prescribing is a complicated decision-making process, undertaken by both patient and doctor, whose intricacies and influencing factors are not yet fully understood [46,47]. By understanding more about influences and the prescribing process, variation in prescribing and the implementation of current best research evidence may be more easily explained. This study aimed to better understand the influences GPs recognize when making prescribing decisions, and why they do not always apply established and well recognized research evidence (such as the evidence within the quality prescribing indicators used by Audit Scotland).

## Methods

We carried out an in-depth ethnographic study of three different general practices in Scotland to explore how practitioners make prescribing decisions and the influences they recognize when making these decisions.

### Sampling and recruitment

Audit Scotland indicators of prescribing quality were applied to a year of PRISMs (Prescribing Information System for Scotland) data (April 2005 to March 2006) to create a sampling frame [48]. We used all nine Audit Scotland quality indicators specific to prescribing in an attempt to give a broad measure of good practice (see Table 1). These indicators included established and well-recognized research evidence and were regularly used by the local health board to identify prescribing quality (discussed further in macro prescribing section). All practices in NHS Tayside (n = 72) were ranked by their performance against all measures. Practices that consistently performed well or poorly on these measures were identified. High ranking practices were selected as practices which performed well were likely to demonstrate good practice and the low ranking practice was likely to provide valuable insights by contrasting practices, processes and values. Two practices ranked as high prescribing quality and one practice ranked as low quality were recruited. The highest ranked practice was atypical, but the research team felt this practice could provide some illuminating findings, so another more generalizable practice was identified to provide comparisons with the low-ranked practice. The low-ranked practice was aware they were not performing well against prescribing measures but agreed to take part to receive comparative feedback. Routinely available data were used to include practice demographics (list size, urban/rural, and deprivation status) in sampling to increase variation and improve the generalizability of the findings and recommendations.

**Table 1:** Indicators of prescribing quality

•	PPI maintenance doses as % of maintenance and treatment doses
•	2.5 mg bendrofluazide as % of 2.5 mg & 5 mg
•	Single diuretics as % of single & combined diuretics
•	ACE inhibitors per 1000 adjusted population per quarter
•	Low dose aspirin per 1000 adjusted population per quarter
•	Statins per 1000 adjusted population per quarter
•	Hypnotics & anxiolytics per 1000 adjusted population per quarter
•	Established antibiotics as a % of oral antibiotics
•	Amoxicillin as a % of amoxicillin and co-amoxiclav

### Data generation

Data generation was carried out between January 2007 and April 2008. The primary research method was non-participant observation, triangulated by interviews and review of practice documentation.

### Observation

AG (social scientist) was present in each practice for three to six months. Practices were observed in sequence and observation was undertaken in a range of locations: GP surgeries with all consenting patients (no patient groups were excluded); home visits; nurse-led clinics; reception; shadowed district nurses, practice managers and pharmacists; practice meetings (both clinical and administrative); practice meetings with Community Health Partnership (CHP) clinical leads; CHP practice pharmacist meetings; and a wide range of informal interactions such as the coffee room, over lunchtime, and tea breaks. Field notes were recorded as soon as possible after each observational period.

### Interviews

GPs and practice pharmacists took part in semi-structured interviews after the observation period. This allowed the findings from the observational research to be fed into the interviews and offered ‘key’ respondents an opportunity to comment. All interviews were facilitated by a topic guide, conducted in the practices, lasted approximately an hour, were audio-recorded, and transcribed verbatim.

### Documentary sources

A number of practice documents were obtained during the period of observation: practice protocols and policy documents; CHP prescribing reports and communication memos/letters; prescribing review reports; news and journal articles; clinical management plans; communication memos; and slides from practice prescribing meetings. There were no substantial differences in the availability or number of documents retrieved in each practice.

Analysis was ongoing, iterative, and was influenced the researcher’s observations in the field as the study progressed. AG and JD met regularly to discuss the emerging analysis. We initially constructed an in-depth ethnographic description of each practice, detailing the practice structure, systems, prescribing processes, communication channels, culture, and values. Field notes were the primary data source. These were supplemented by appropriate practice documentation and interview data. Practice documentation was scanned and/or synopsis written of the relevant points and issues. Data were imported into Atlas.ti 5.5 coded and organized into patterns and categories. JD carried out double coding on a sample of data, and discrepancies were discussed. An interpretative and constant comparative approach was used to explain and understand the similarities and differences between the three practices [49]. Memos were written throughout the analytic process and provided a detailed account of thought processes and amendments as new data was added. Relevant helpful conceptual and theoretical frameworks were drawn from; Gabbay and le May’s mindlines [41], the notion of identity from Weick’s organizational sensemaking [50], and Sheaff *et al.*’s soft governance [51,52].

This ethnographic study was approved by the Tayside Committee of Medical Ethics B (06/S1402/99).

## Results

The findings are from 394 hours of participant observation, nine semi-structured interviews and a review of 46 practice documents in three general practices in NHS Tayside, Scotland. These practices have been pseudonymized as Rubain, Balla, and the Haun.

Disentangling the relationship between long-term prescribing behaviors, the flux of change from regular reorganization, and the clamour of daily practice activity was analytically complex. Despite these tensions, patterns of prescribing behavior were strongly evident in the data. We found the high-ranking practices made two different types of prescribing decision; macro and micro, whereas the low-ranking practice made micro-only decisions. Macro prescribing involved strategic policy decisions whereas micro decisions were made about an individual patient. This paper presents these prescribing decisions and explores the differences between the high-ranked practices and the low-ranked practice. This paper begins with a table (Table 2) detailing the practice characteristics and a description of the different practice narratives and values, followed by a detailed analytic discussion of these prescribing patterns.

**Table 2:** Practice characteristics

	**Rubain**	**Balla**	**The haun**
**No. of GPs**	3 f/t & 1 p/t	1 f/t & 1 p/t	2 f/t & 5 p/t
**Prescribing quality Rank**	4/72 (high)	1/72 (high)	71/72 (low)
**List size**	6000	2000	8500
**% Population** in most deprived deprivation category quintile*	0.03	1.93	20.58
**Location**	Market town	Rural	Urban
**Practice pharmacist**	2.5 days a week	0.5 day a week	3 days a week

*Data from ISD Scotland: Practice populations by deprivation status as at 30th September 2006.

### Practice narratives and values

Each practice had a narrative about their organization, communication strategies, and their values. These narratives were co-created in Rubain and the Haun and were dominated by the lead GP in Balla. Their narratives were an important part of their practice identity, ‘what kind of practice are we?’ [53]. This identity shaped practice norms: how the practice was organized, how they communicated, worked as a team and interpreted and implemented guidelines likely to improve prescribing quality.

### Rubain

Rubain staff were proud of being an organized, efficient, holistic, and accountable practice that provided a high-quality service to their patients. They viewed prescribing as an integral part of their service and included cost efficiencies in their definition of quality prescribing. They valued teamwork, cohesiveness, shared decision making, consistency in their prescribing behavior and competition (*e.g.*, prescribing indicator reports comparing practices in the CHP). Rubain’s values led them to organize regular face-to-face meetings, have tight protocols and systems (*e.g.*, to maintain relationship continuity of care), employ two practice managers, and utilize their information technology (IT) system to implement decisions.

### Balla

Balla staff were proud of being organized, efficient, holistic, and providing the best possible service to their local community. They were pleased they were able to offer patients an on-the-day appointment. High-quality prescribing, not including cost efficiencies, was viewed as integral to providing a high-quality service. Balla valued simplicity, creativity, innovation, and change. Balla’s values of innovation were instigated and implemented by the lead GP who regularly invested time thinking about how the practice’s systems and processes could be improved. This GP valued effective, simple processes, and systems that he felt allowed him more time to focus on quality of care and prescribing.

### The Haun

The Haun were proud of their modern, egalitarian practice, which valued part-time working and relied on IT communication strategies (email and clinical system). The high number of part-time staff meant the practice struggled to provide relationship continuity of care, despite all practitioners viewing this as important. The Haun collectively said they valued clinical autonomy and tended to shy away from formal and tight organizational processes and systems. However, some GPs admitted they felt the practice should move towards a more system-based approach and retain their egalitarianism. The findings presented are their collective narrative rather than ‘confessions’ from those prescribers who chose to build a closer rapport with the researcher.

### Two different prescribing decisions

Macro decisions were collective; strategic prescribing policy decisions which considered the average patient, one disease/condition, and were focused on one drug. Influenced by research evidence (primarily guidelines) and clinical governance mechanisms (practice pharmacist, health board formulary, comparative prescribing indicator report). Thus, macro decisions were based on population-level data about specific groups and largely ignored contextual and patient level factors. The high-ranking practices interpreted this information in light of their population and values and formulated a prescribing policy. Macro prescribing required research evidence decisions but also communication mechanisms and interest to convert this policy into practice.

Micro prescribing decisions were made during a consultation considering patient views, preferences, circumstances and frequently more than one disease. When making micro decisions, all GPs relied on internalized personal formularies and clinical judgement, termed ‘prescribing mindlines’. Neither the high- nor low-ranking practices engaged with research evidence at this stage.

These decisions were made in different contexts, with different influences and different people involved. They could operate independently, but evidence-based, high-quality prescribing seemed to require a mechanism for formulating and transferring the macro into micro and thereby, into practice. Effectiveness of these decisions was mitigated by practice organizational structure and communication processes. This point will be addressed below.

### Micro prescribing

Micro prescribing decisions were made by all clinicians. Micro prescribing was about trying to apply scientific evidence to the individual patient considering the patient’s preferences, values, and circumstances, and in many cases, other conditions. This observational data found prescribers used ‘mindlines’ to make these decisions and confirmed their use specific to prescribing [41].

### Prescribing mindlines

These were personal formularies developed from and informed by their experience of medication (including patient’s experiences), specialist advice, discussions with their practice pharmacist and GP colleagues, and the practice’s macro prescribing policy (if present). GPs rarely looked up information about medicines and relied on these prescribing mindlines. For example:

‘I mean things are definitely habitual, I always prescribe a particular drug, those I know off the top of my head, why would you prescribe an alternative that you didn’t know so much about’. (GP 2 interview, the Haun)

GPs relied on personal experience and social networks to update their mindlines. These influences are now discussed in turn.

### Experience of medications

Every GP explained they placed their experience of medications as their most valued and strongest influence over prescribing. Experience was gained over time, through medical training, years in practice, and exposure to a number of different prescribing decisions and their outcome. By prescribing drugs they knew well, GPs could make expeditious prescribing decisions. In the majority of consultations observed, GPs tacitly ‘knew’ what to prescribe. Differences were observed when the GP was initiating medication or looking after a patient on medication they did not know well. In this extract from field notes, the GP switches the patient from a regular unfamiliar therapy to another that they more often prescribed:

‘The patient who has entered the consulting room has just registered with the practice so there are no notes. The patient comes into the consulting room and immediately tries to tell the GP how he has been feeling, he struggles with this and describes the circumstances by which these symptoms have occurred in the past. The GP asks this patient a series of questions and then asks him if it is ‘vertigo or dizziness’ and the patient says ‘no’. The patient tells the GP he is on Stelazine and the GP looks up the British National Formula (BNF) to see if there are any side effects. The GP prescribes Prochlorperazine without referring to any guidelines.’ (Rubain, fieldnotes, 14.02.2008)

All the GPs reported bad patient experiences with specific prescribed medication that influenced their opinion of those drugs and thus influenced their future prescribing behavior. With drugs they knew well and commonly prescribed, one patient’s bad experience did not immediately influence their opinion.

### Secondary care

All the GPs referred to secondary care as one of the strongest influences over their prescribing. Secondary care was defined in this study as ‘care that is provided by specialists or other healthcare staff working in the clinic upon referral from a GP’. GPs followed prescribing advice from specialists. This communication was primarily by letter or discharge note, frequently hand written only stating the medication that should be prescribed.

GPs explained following secondary care recommendations was often how they gained experience of new medications. They read and learned more about the medication to adequately look after patients. As they looked after more patients on the medication they gained experience:

‘There’s been new drugs that have come on-board and the way they’re being used is according to the local specialist and how they prescribe’. (GP 2 interview, Rubain)

GPs became aware of what specialists were prescribing for a particular disease and came to follow this trend, as this extract from observation illustrates:

‘The GP prescribed Metformin for a patient who was overweight and had polycystic ovaries (PCO). After the consultation I asked about this. The GP explained that ‘Metformin has been prescribed for the past few years by gynaecologists. This patient has been trying hard to lose weight with little success so her PCO could be why she is not losing weight, so it is a good excuse to try it and see how she gets on”. (Balla, fieldnotes, 20.03.2007)

In Rubain and Balla the influence of specialists was the strongest reason why they deviated from prescribing policy.

GPs started to notice trends in secondary care prescribing, and if they felt comfortable and had come to know the medication they emulated this. Thus, prescribing initiated by specialist recommendation was an important part of the iterative development of prescribing mindlines. Hospital-initiated prescribing practices eventually entered a GP’s own mindline through familiarity.

### Interactions with colleagues

In all practices colleagues were valued and trusted as important sources of information: sharing knowledge, experiences and asking advice. Colleagues were from within the practice, other GPs working locally, and specialists.

Information sharing was regularly observed in all practices at prescribing meetings, in the coffee room, and one-to-one in their consulting rooms. Within each practice, a number of GPs had areas of special interest and were asked for advice. GPs also reported discussions with other GP colleagues at external practice events, such as continual professional development meetings (CPD).

The quote below illustrates the influence colleagues had on prescribing:

‘I guess the other doctors in the practice. I mean if you are, if I have got a difficult problem I will take it to my other doctors and take advice about what they would do. Seeing what colleagues have done when you look at the notes, somebody is prescribing this or somebody treats that why, what comes out of hospital, hospital doctors recommend’. (GP 1 interview, the Haun)

Regular daily conversations in Rubain and Balla involved clinicians discussing or sharing work but also narratives about patients. The GPs in these practices used the coffee room at lunchtime specifically to be available for discussions. Rubain also had a morning meeting where they divided up home visits and liaised with reception and practice managers. The organizational features of the Haun meant collective informal discussion was more difficult, so they tended to have these conversations one-on-one, with trusted others behind closed doors.

When apprehensive about a prescribing decision, they sought advice and/or reassurance from colleagues. On occasion, these narratives were about letting off steam, for example:

‘Dr A is expressing his frustration at Dr B about a problem he is having getting medication for a patient. The Dr prescribed a medication but the village pharmacy does not stock it. The GP then contacted the specialist who suggested something else but the pharmacy does not stock this either’. (Rubain, fieldnotes 28.02.08)

The GPs in all practices asked their pharmacist for advice on individual patients, advice on dosing, interactions, or suggestions about what to prescribe. Practice pharmacists searched the relevant literature and the national formularies (BNF and Martindale’s Pharmacopoeia). Practice pharmacists were employees of the local health board and based in practices to improve prescribing (further information in the macro prescribing section Page 11):

‘People who are on drug interactions or side effects from the drugs, she is very good at, if we’ve got queries about drugs or dosages or interaction she’s very good at that’. (GP 3 interview, the Haun)

Colleagues have tacit knowledge that can be difficult to retrieve from other sources. Conversations in communal spaces ensured these were open to all prescribers. These interactions resulted in the iterative development and modification of prescribing mindlines. Through regular communication and discussions in Rubain and Balla, we hypothesized prescribers developed shared prescribing mindlines but retained a degree of individual interpretation based on their personal experience, preferences, and values. In the Haun, the large number of part-time staff and lack of face-to-face communication mitigated the collective sharing of stories and mindlines, contributing to low prescribing quality.

### Drug representatives

All practices played down their engagement with drug representatives, though all did occasionally use them to provide a ‘free’ lunch. Drug representatives were present in Balla and the Haun approximately once a month and on a more *ad hoc* basis in Rubain. In Balla, the second GP felt she did not have time to keep up-to-date so would see representatives. All GPs reported viewing the representatives with scepticism:

‘They used to be more of an influence in the old days. They don’t tend to influence things so much these days because we always tell them that for any change in our prescribing it would have to be discussed with (pharmacist named) and so they should speak to (pharmacist named). You do tend also to find that they tend to all be promoting the same type of medicine anyway’. (GP1 interview, Rubain)

As prescribing trends in PRISMs data were difficult to observe, it was impossible to measure the effect of a drug representative visit on each practice. Although information from drug representatives was likely to have a subtle effect on GP’s prescribing mindlines, no consultations were observed where the promoted drug was prescribed subsequent to the representative visit.

### Internalization of information at the micro level

At the micro level, evidence and information relevant to prescribing was internalized through applying it to patients. GPs observed how patients responded to medication initiated in secondary care. When there was little alternative, GPs tried a new drug or dose. If the patient responded well, GPs would try this in another patient with similar symptoms and risk profile. GPs observed how patients responded to this medication by engaging in closer monitoring and follow up. Discussing this with colleagues was another important mechanism for the internalization of evidence at the micro level.

### Applying prescribing mindlines

Prescribing mindlines were applied to individual patients considering their preferences, values, and circumstances. GPs and patients did not engage in shared decision making about whether to prescribe or not and rarely in the choice of preparation. Patients were observed actively being involved in decisions about the method of administration, such as capsules instead of tablets. Although patients were not interviewed, they appeared to be satisfied with this level of involvement.

### Macro prescribing

These population-based decisions were influenced by guidelines and clinical governance and were shaped by practice values, organization, and communication channels.

### Soft governance mechanisms

Health Boards and Community Health Partnerships (CHPs) are accountable in Scotland through clinical governance to ensure prescribing is evidenced based and cost effective. GPs have independent contractor status, therefore cannot be managed by traditional command and control mechanisms. Sheaff *et al*.’s work illustrated the fact that CHPs use a range of soft governance mechanisms to influence prescribing [52]. These techniques try to appeal to professional values and build relationship and rapport between the CHPs and practices [51]. By doing so these approaches are dependent on how practices legitimized them [52]. Each soft governance mechanism witnessed is discussed in turn below.

### Health board prescribing formulary

The health board formulary is a guide of recommended drugs intended to direct choice towards a rational selection of drugs based on clinical efficacy, safety, patient acceptability, and cost-effectiveness. Rubain and Balla modified this formulary to suit their preferences and experience by developing their own formulary. Rubain set reminders on their clinical system to reinforce this. Practice formularies listed fewer drugs than the health board formulary. This allowed practices to retain some clinical autonomy, yet rationalize and standardize their prescribing. The Haun did not place such value on consistency; they tended not to discuss what they prescribed and trusted individual professional opinion:

‘I have to say I think that prescribing policy is something that perhaps we could be tighter on in this practice, we don’t really have a formal, this is the drug that we use for this situation in this practice, we tend to prescribe what we want to prescribe ourselves as individuals’. (GP 1, interview, the Haun)

### Prescribing indicator report

CHPs produced a prescribing report, which presented practices ranked by their performance against quality and cost-efficiency indicators applied to PRISMs data, with the intention to motivate practices by peer comparison. Rubain legitimized these performance indicators and responded to this sense of competition. At their prescribing meetings, they collectively reviewed their performance and identify areas for audit. They had been a fundholding practice, and the research team related their experience at critiquing prescribing to this:

‘We have meetings with (pharmacist named) once every four weeks, the last Friday of the month. First of all, we look at, because of our prescribing, a monthly update in terms of how our prescribing compares with the CHP average and so on and he recommends changes to our prescribing patterns’. (GP 1 interview, Rubain)

Balla and the Haun did not value the prescribing report due to the limitations of PRISMs data. PRISMs measures cost and volume and is not linked to case-mix. These practices had older and younger populations, respectively, than the health board and national averages and felt this skewed their data.

### Practice pharmacists

Practice pharmacists were the most sophisticated and legitimized of the soft governance mechanisms. They were champions of rational prescribing, building relationships and rapport, and legitimizing CHP policy and priorities by appealing to relevant practice values. They were employees of the health board but based within practices to facilitate rapport, legitimization and improve prescribing.

The role of practice pharmacists was very distinct in comparison to traditional community and hospital pharmacy positions. They were responsible for disseminating and implementing CHP prescribing policy (both quality and cost) and new guidelines; conducting clinical audits; and giving prescribing advice. The latter two roles were part of rapport and relationship building to facilitate implementation of CHP policy. Due to varying degrees of legitimization of CHP policies, managerial forms of control and the practice organization the roles and responsibilities of each pharmacist varied:

‘Because our roles have generally evolved rather than being dictated. They tend to evolve within the practice depending on what the practice priorities are and I suppose the interests of the practice pharmacist and the GPs as well’ (Practice pharmacist interview, the Haun)

In Rubain, their pharmacist adopted a strategic role spending most of his time influencing the practice’s prescribing policy. He helped them keep up-to-date with the evidence, constrain the cost of their prescribing, and conducted medication reviews. In Balla, their pharmacist had an operational role where she carried out the practice’s warfarin clinic, conducted the occasional medication review, and provided advice on an individual patient basis. In the Haun, their pharmacist carried out an operational and strategic role. She was involved in filtering evidence and CHP policy into the practice, but because the practice did not have a prescribing policy her effect was limited to her operational role (audits, medication reviews, processing and checking secondary care recommendations, and providing advice).

The pharmacist’s strategic role was viewed as a prescribing leadership role by the two larger practices. The Haun and Rubain relied on their practice pharmacist to inform them of the latest evidence (usually guidelines). The pharmacists had a preferred hierarchy of guidelines, with Scottish Intercollegiate Guidelines Network (SIGN) guidelines first and foremost, followed by National Institute for Health and Clinical Excellence (NICE), and local health board and CHP guidance. GPs felt they did not have sufficient time to keep up-to-date and to reflect on new evidence. The pharmacists filtered evidence through prescribing meetings that also facilitated the dissemination of CHP policies. In all practices, the GPs trusted what the pharmacist said and did not question the sources, but they did challenge some of the CHP cost-saving initiatives.

Only in Rubain was the pharmacist able to fully endorse CHP policy, due to them valuing cost-efficiencies. In the Haun and Balla, pharmacists were limited in their ability to persuade GPs due to organizational constraints. In Balla, the pharmacist was only in the practice one morning a week and spent much of this time running the practice’s warfarin clinic. Also, the lead GP liked to keep on top of the evidence himself, limiting opportunity for their pharmacist. The pharmacist did distribute CHP policies, but due to her limited time she was unable to engage in face-to-face discussion. In the Haun, the high number of part-time staff and lack of collective face-to-face communication limited her effect.

Practice pharmacists were seen to have specialist skills in interpreting guidelines and CHP policies in light of practice populations. The pharmacists were able to audit and interpret this information in the clinical system data. Implementing macro prescribing policy involved identify the numbers of patients affected, gauging whether they needed to call them in for a medication review or opportunistically review and not start any new patients on the drug and/or dose.

### Prescribing meetings

Prescribing meetings in Rubain and the Haun had different atmospheres to reflect their different values towards managerial forms of control and consistency in their prescribing behavior. In Rubain, these meetings were generally punctually attended by all clinical staff. The practice pharmacist presented the information in detail by PowerPoint presentation, that were actively discussed:

‘First of all … a monthly update in terms of our prescribing compared with the CHP average and so on and he recommends changes to our prescribing patterns. Obviously usually with evidence based in terms of recommendations from NICE or SIGN guidelines or whatever, and we discuss it and almost invariably we agree with what he says’ (GP1 interview, Rubain)

Whereas in the Haun, these meetings were poorly and not punctually attended (they were also not regularly scheduled). The pharmacist’s agenda was on various pieces of paper (many scrap) and the GPs accepted her recommendations. The informal nature to these meetings fitted well with the organizational culture of the practice rather than a lack of interest by the GPs. The practice was going through a period of turbulence, and business issues were dominating. Regardless, without a prescribing policy and formulary there was less need for discussion, and the practice pharmacist was limited in her effect. These meetings were used by the GPs as a means of keeping up-to-date with the evidence.

In Balla, they had more general clinical meetings where prescribing policy was discussed, unfortunately due to the pharmacist’s limited time at the practice she was unable to attend.

### Why was macro prescribing not taking place in all practices?

There were differences in practice identity, characteristics, and organization that influenced adoption and implementation of macro prescribing policy and variation in their prescribing practices.

Rubain and Balla were systematic and organized. The Haun was also coming to increasingly value being organized and was undergoing a culture shift towards tighter processes and systems during the period of observation as a result of the New General Medical Services (nGMS) contract.

Rubain and Balla were proactive, holding meetings to anticipate change and to plan, devising procedures, and ensuring resources were in place. For example, Balla planned extensively for a change of IT system, learning from other practices but also brainstorming to meet their needs and circumstances and minimize impact to their daily work. The Haun was going through a period of turbulence with business issues dominating formal meetings. The Haun was a reactive practice; they did not invest the same amount of resources to planning and organizing clinical systems and processes. The quote below illustrates this point;

‘I think it is, you do things the way you do them because it’s the way you do them and you are used to doing them that way, and I think sometimes it is hard to take a step back and look and see what you could be doing differently, because you just assume that you are doing things and it is working well, and it is not until there’s a problem that you think well we should really have had a system to try and stop that problem happening, if only we’d been doing this and this, this would never had happened. But until, I think you just plod along, you are busy doing other things, you don’t have a lot of time to sit and think about it’. (GP 1 interview, the Haun)

The two practices that performed well against the prescribing measures both had leadership; both Rubain and Balla had clinical and administrative leadership, however in Balla these roles were performed by the lead GP. The Haun was an egalitarian practice, with no clinical leadership and the practice manager heavily involved with managing reception taking her away from managing the GPs and other organizational aspects of the business. Leadership was important for practice organization and managing change. Practices were constantly being forced to manage change with reviews of policy and practice from CHP, HB, and Government, and through modifying behavior and practice to be in line with the latest evidence-based medicine (EBM). Leadership involved ensuring these issues were addressed and dealt with. In Rubain and Balla, a GP would oversee the clinical and organizational aspects, albeit with paternal and autocratic leadership styles, respectively. These clinical leaders ensured issues were addressed, would drive change, and be involved in co-ordinating the practice organization. Rubain and the Haun also had practice managers who were important in leading the organizational aspects. Rubain had invested heavily by employing two practice managers and in the Haun, due to the high number of part-time staff (both clinical and administrative), the practice manager spent a considerable amount of her time organizing reception, rotas, and the day-to-day practice management.

The two higher-ranked practices had strong organizational cultures and practice identities that valued and reinforced organization control, with which macro prescribing policy fits well. The Haun struggled to organize collective meetings with a large percentage of their clinical staff working part-time, with business issues dominating the practice agenda. The Haun did not have a strong organizational culture or identity; operating primarily as a reactive practice. In the Haun, formulating a macro prescribing policy was more difficult with their patient mix, and trying to ensure consistency in prescribing was not a high priority at the time of observation.

Prescribing decisions are context dependent, so each practice had to interpret the evidence to suit their local population. Rubain’s population was mixed, Balla’s population was elderly, rural, and affluent, and the Haun was responsible for a young, urban, deprived population. The practice populations affected each practice’s culture and organization. In Balla, they looked after patients who were generally compliant and with little social problems in comparison to the Haun who cared for a larger number of patients with psychosocial problems.

Some GPs in Rubain and Balla were happy to let consultations overrun on the basis of addressing concerns and educating patients would save time in the future. GPs in the Haun were not as relaxed about their surgeries over running. This practice struggled to maintain high levels of relationship continuity and was in a central urban location with no patient car park and only short stay fee parking nearby.

There were a large number of factors that influenced practice adoption and implementation of macro prescribing policy. The Haun illustrated prescribing issues may not be a practice’s top priority and organizing for efficient, effective, and evidence-based prescribing required practices to be proactive and plan in prescribing meetings with all GPs present, where possible. There were a number of confounding factors that made this task more difficult, such as a high number of part-time staff and a deprived population.

### Micro and macro prescribing model

We developed a model to illustrate the influences recognized by practitioners in the high-ranking practices, how and where they filter into prescribing, and the interaction between macro and micro prescribing.

In the high-ranking practices new evidence was discussed and practice-prescribing policy (macro prescribing) was modified, if necessary. Rubain valued cost efficiencies and legitimized CHP soft governance mechanisms, so CHP policy was also filtered into the practice, along with new evidence by their practice pharmacist.

This model includes ‘other quality improvement (QI) efforts’ as GPs reported these in informal conversations with the researcher, however this was only observed once. GPs reported taking part in research studies at the local university and reported presentations by specialists at CPD meetings.

**Figure 1:**
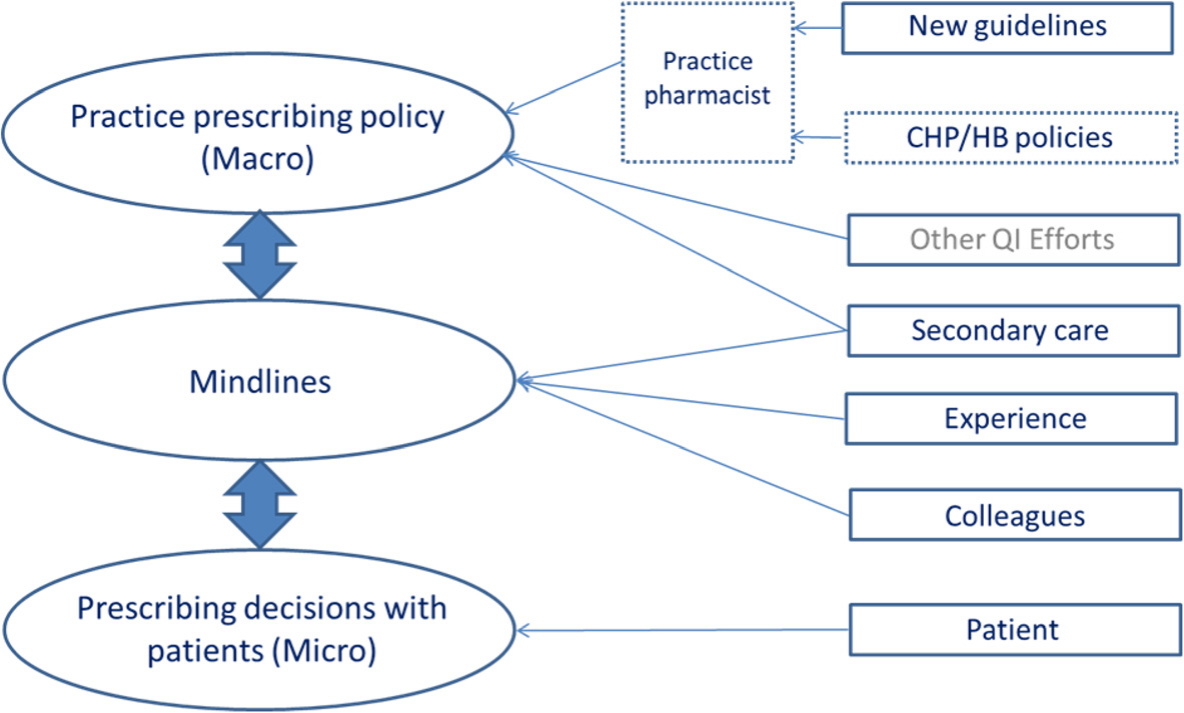
High-ranking practices prescribing model (Rubain and Balla).

Because macro prescribing policy was missing in the low-ranking practice, the influences down the right-hand side of the model were primarily filtered into practices via their personal mindlines on an *ad hoc* basis (Figure 1).

## Discussion

This study found the high-ranking practices continuously made and applied both macro and micro prescribing decisions, whereas the low-ranking practice only made decisions at a micro level. These findings suggest macro prescribing is required to inform the implementation of research evidence at the micro level. This distinction raises important implications for current quality improvement strategies and the EBM movement, and suggests the need to focus on cultural change in some practices.

The main difference between the high and low-ranking practices was the formulation of a macro prescribing policy. Quality improvement strategies are advocating consistency and standardization at the practice level based on research evidence, shifting evaluation, and management decisions away from the individual doctor [54]. Macro prescribing decision-making needs to be supported by organizational processes and systems for consistent implementation. Good teamwork [55-57] and effective communication channels are a key part of providing high-quality care [58,59]. Strong mechanisms and processes for transferring information and developing shared meanings for action are crucial [60,61].

Most decisions to prescribe drugs involve a combination of factors [18]. Research evidence and written information is only one influence [1,32]. Colleagues are important social influences [1,30], illustrating individual and organizational learning can take place at the same time [62] but requires a well-functioning team [56,63]. Experience shapes the way research evidence influences clinical practice [64,65]. All these factors contribute to the development of mindlines, which ultimately influence prescribing decision making [41,66] at the micro prescribing level.

Engagement with EBM and clinical governance mechanisms took place at the macro prescribing level. Practice identity, organization, and culture can be barriers to engagement with EBM and quality improvement mechanisms. Practices need to engage with the evidence and interpret their practice data to make improvements. To do this they need to be organized with practice processes and systems in place that support macro prescribing. Practices need to value macro prescribing and consistency in behavior across all GPs for this to be effective.

Practice pharmacists were essential in the larger practices to filter research, provide prescribing leadership, and interpret the evidence in light of the practice population. This finding is relatively novel because there has been little research into the influence and effect of pharmacists working in general practice. Balla practice, however, shows a pharmacist is not always essential. This may be due to their small size, their investment in system refinements, or due to the clinician’s personal engagement with research evidence. Without a pharmacist, practices need to find another mechanism to filter and interpret evidence to inform macro prescribing and lead to a consistent change in (micro) practice.

Our study found another important difference between the high- and low-ranking practices was their collective use of the coffee room at certain times, such as lunchtime. Colleagues were an important source of information [1,67] in all practices however, having these conversations in shared spaces rather than one-to-one allowed others to contribute and learn [41]. The low-ranking practice had a high number of part-time staff and a lack of face-to-face communication. By organizing the practice to have all practitioners in the practice on the same day could facilitate informal and formal collective discussions.

This work builds on and supports the existing body of literature in the range of influences recognized by GPs when prescribing [1,15,18-20,22,41,68-70], and explores these influences in light of more recent health service reforms, such as the nGMS contract [71]. This is the first study we are aware of which holistically explores all influences recognized by GPs when prescribing and describes how they have embedded these influences into routine practice. Current prescribing quality improvement interventions tackle prescribing at the macro prescribing level [11,72-77]. So those practices that are not organized to integrate macro prescribing are unlikely to perform well against quantitative measures of prescribing quality. The ubiquitous use of electronic medical records in primary care and the advances in data extraction and linkage offer huge opportunities to measure and establish variation at the micro prescribing level. This can be seen in the growing use of informatics interventions to identify patients at high-risk of an adverse drug event [78,79].

Many of the interventions to influence prescribing have been perceived by the medical sociology community as threats to GPs clinical autonomy [20,21,30]. In this study, soft governance mechanisms were welcomed at the macro prescribing level as a means to deal with the overwhelming amount of evidence targeted at GPs, but these findings support the place of clinical autonomy at the micro level [31].

Patient perspectives and expectations may have been expected to play a significant role at the micro level, but they did not emerge as a major influence on the choice of preparation (*e.g.*, diclofenac or paracetamol). Although prescribing mindlines were flexible enough to consider the patient’s preferences, values, and circumstances, the GPs and patients rarely engaged in shared decision making about choice of medication. GPs and patients did engage in treatment decisions about choice of administration method (such as oral or topical). This finding is consistent with the published literature [80,81].

### Strengths and limitations

By using ethnographic methods, this study was able to explore routine prescribing practices within three general practices. The strength of this work was the number of hours of observation, in carefully sampled multiple sites, by a researcher who was not a healthcare professional. This allowed consideration of factors that may be taken for granted through an interview study or by someone with a healthcare background. As life in general practice is constantly changing this is a snapshot of prescribing practices in one health board in Scotland. It is hoped some of these findings will resonate with GPs and primary care providers in other contexts. Tayside Health Board has invested in practice pharmacists more heavily in comparison to many other health boards, thus some insights may not be directly transferable to other settings. Due to the small number of practices included, this study will also not represent the full range of views and influences that exist. The practices that took part were ranked by prescribing quality indicators. These indicators do not represent the full range of characteristics GPs may attribute to high-quality prescribing, being a good practitioner, or having an effective organizational prescribing culture.

### Implications for policy and practice

Practices are likely to benefit from recognizing both different types of prescribing decisions we describe. Practice identity appeared instrumental in shaping engagement with EBM and clinical governance, and influenced practice organization and communication channels. Organization and communication channels were also key influences on macro prescribing and quality improvement.

The EBM movement requires a shift from tacit knowledge to clinical practice grounded in data [64]. Practice pharmacists had an important role feeding new evidence into practices, interpreting this in their prescribing data and translating in the context of changing evidence. Recent improvements in prescribing in Scotland may be partly due to pharmacist involvement [82]. Leadership was key for interpretation and co-ordinated practice engagement. Practice meetings were invaluable to prescribing sense making, macro prescribing policy, and implementation. Practice pharmacists are not a luxury enjoyed by all, however; practices may themselves need the skills to interpret practice data in the light of new evidence and collectively modify macro prescribing policy, and this will require time.

Clinical judgement is an important part of prescribing behavior at the micro prescribing level. Macro prescribing and EBM are difficult to apply in the context of patient co-morbidities and polypharmacy. At the micro prescribing level, GPs used prescribing mindlines, developed, reinforced, and shared with colleagues. Practices should be aware of how macro prescribing influences their prescribing mindlines and utilize this.

Currently quality improvement focuses on prescribing at the macro level. Further work and interventions targeted at the micro prescribing level are required, such as the Data Driven Quality Improvement in Primary care (DQIP) project. This is an informatics-based prescribing quality improvement intervention currently being tested in a randomized controlled trial that uses macro prescribing policy to influence micro prescribing [78]. DQIP applies newly developed prescribing safety measures [83] to practice prescribing data and feeds this back to practices in a manner that prompts practices to review patients at risk due to a range of factors such as age and co-prescribing.

## Conclusion

This research has illustrated practices make two different kinds of prescribing decisions, and the influences upon these decisions vary. Although micro prescribing will operate at the time of clinical decision making during consultations, evidence-based, high-quality prescribing seems more likely to occur when there is a functional macro prescribing policy. Macro prescribing policy seeks to ensure prescribing decisions are based on evidence and applied consistently where possible. Macro prescribing offers a framework from which GPs deviate when deemed appropriate. Macro prescribing informs the micro and without it, the low-ranking practice had no mechanism to reflect on the evidence in light of their practice population and internalize this information to inform their prescribing mindlines and thereby practice.

There is a need for practices to recognize these different prescribing decisions and influences and their interdependent relationship. Currently, policy makers and the EBM movement focus on assessing prescribing in aggregated micro level data [84]. With this focus, judgements are erroneously made about macro prescribing decisions. Current prescribing quality improvement initiatives that target macro prescribing pay insufficient attention to the delivery and implementation of best research evidence at the micro prescribing level. This may explain the small effect sizes of prescribing quality improvement interventions to date. General practices with lower prescribing quality are likely to benefit from support with organizational processes to support macro prescribing. Only then they will be able to refine this and improve prescribing for individual patients.

## Abbreviations

GP: General practitioner; NHS: National health service; nGMS: New general medical services contract; PRISMS: Prescribing information system for Scotland; CHP: Community health partnership; BNF: British national formulary; CPD: Continual professional development; PCO: Polycystic ovaries; NICE: National institute for clinical excellence; SIGN: Scottish intercollegiate guidelines network

## Competing interests

All authors declare no competing interests with this work.

## Authors’ contributions

AG was funded by the Scottish Government Health Directorates Chief Scientist Office on a PhD studentship (CZS/1/43). JD and FS were AG’s supervisors. JD is responsible for the conception, contributed to the study design and analysis of data and commented on drafts of the manuscript. FS provided academic supervision and commented on drafts of the manuscript. AG is responsible for the study design, data collection and analysis of data. AG prepared the first manuscript and is responsible for this article. All authors read and approved the final manuscript.
